# Stent Scraping for Histology: An Alternative Method for Obtaining Tissue to Rule out Neoplasia

**DOI:** 10.1155/DTE.1.107

**Published:** 1994

**Authors:** Jerome H. Siegel, Annamali Veerappan, Romulo Prudente, Franklin E. Kasmin, Seth A. Cohen

**Affiliations:** 1 Sections of Endoscopy and Pathology Beth Israel Medical Center North Division New York NY USA; 2 F.A.C.P. F.A.C.G. 60 East End Avenue New York NY 10028 USA

## Abstract

Despite improvement in diagnostic modalities, confirmation of a histologic diagnosis of cancer of the biliary tree and pancreas remains elusive. Attempts to collect positive cytology specimens from vigorous brushings or washings obtained at endoscopy or percutaneously are often unsuccessful. In our unit, we have increased the yield by obtaining tissue scraped from prostheses that have been previously placed in either the bile duct or the pancreatic duct. The stents are first flushed with saline to collect cytology specimens, after which, they are bisected and scraped, and these contents are prepared in a manner similar to that used to prepare biopsy samples. Twelve of 16 scraped samples, 9 bile duct and 3 pancreas, were positive for adenocarcinoma. The cytology specimens were positive in only 4 of the 12. We recommend this method of sampling from material contained within prostheses as an adjunct when previous brushings, washings, or biopsies are negative.


*Diagnostic and Therapeutic Endoscopy*, Vol. 1, pp. 107–112
Reprints available directly from the publisher
Photocopying permitted by license only

© 1994 Harwood Academic Publishers GmbH
Printed in Malaysia

### Stent Scraping for Histology: An Alternative Method for Obtaining Tissue to Rule Out Neoplasia

JEROME H. SIEGEL, ANNAMALI VEERAPPAN, ROMULO PRUDENTE, FRANKLIN E. KASMIN and SETH A. COHEN

*Sections of Endoscopy and Pathology Beth Israel Medical Center North Division New York, NY*

*(Received August 4, 1993; in final form February 3, 1994)*

Despite improvement in diagnostic modalities, confirmation of a histologic diagnosis of cancer of the biliary tree and pancreas remains elusive. Attempts to collect positive cytology specimens from vigorous brushings or washings obtained at endoscopy or percutaneously are often unsuccessful. In our unit, we have increased the yield by obtaining tissue scraped from prostheses that have been previously placed in either the bile duct or the pancreatic duct. The stents are first flushed with saline to collect cytology specimens, after which, they are bisected and scraped, and these contents are prepared in a manner similar to that used to prepare biopsy samples. Twelve of 16 scraped samples, 9 bile duct and 3 pancreas, were positive for adenocarcinoma. The cytology specimens were positive in only 4 of the 12. We recommend this method of sampling from material contained within prostheses as an adjunct when previous brushings, washings, or biopsies are negative.

**KEY WORDS:** brush cytology, biopsies, cholangiocarcinoma, ERCP, stents (prostheses)

### INTRODUCTION

Cancer of the pancreas and biliary tract, both primary and metastatic involving the extrahepatic and intrahepatic ducts, is often difficult to diagnose despite developments in various imaging and endoscopic procedures such as endoscopic ultrasound (EUS), computed tomography (CT), magnetic resonance imaging (MRI), endoscopic retrograde cholangiopancreatography (ERCP), and peroral cholangioscopy (Nix *et al.*, 1988; Snady *et al.*, 1992; Siegel, 1991). Obtaining a tissue diagnosis for these cancers without surgical intervention is often difficult (Muro *et al.*, 1983; Kawai *et al.*, 1987; Harada, 1979; Endo *et al.*, 1974; Hatfield *et al.*, 1976; Roberts-Tomson and Hobbs, 1979). The reasons for reduced tissue yield include: 1) the small size of the tumor; 2) the fibrotic and scirrhous nature of these lesions; and 3) an associated desmoplastic reaction. A tissue diagnosis is essential before initiating definitive therapy for a neoplasm, such as chemotherapy and radiation, despite a high index of suspicion.

Herein, we describe a technique we use to obtain tissue from occluded stents, which were removed for exchange, and we confirmed the diagnosis of cancer in 12 of 16 patients. Two case reports will be presented to illustrate the technique and the significance of carefully examining stents, that were removed from either the bile duct or pancreatic duct, for tumor tissue.

Address for correspondence: Jerome H. Siegel, M.D., F.A.C.P., F.A.C.G., 60 East End Avenue, New York, NY 10028.

### PATIENTS AND METHODS

Sixteen stents removed from 16 patients, (9 men, 7 women, mean age 65 yrs., age range 47–83 yrs.) 11 bile duct, and 5 pancreas, were examined, and specimens were extracted and prepared from the stents as described in the Methods. This prospective analysis was conducted over a period of 17 weeks, from January to April, 1993. The stents had been placed from 3 to 5 months before the patients returned for stent exchange. The patients returned because of symptoms related to stent occlusion, i. e., cholestasis, low grade fever, jaundice, biliuria, and pruritus. None became septic before exchange. Bile duct stents, 6 11.5 Fr., 5 10 Fr., were placed to ameliorate symptoms of jaundice that were secondary to a stricture of the bile duct. There were 3 Klatskin type III lesions, 4 Klatskin type II and 4 Klatskin type I. Pancreatic stents, 2 10 Fr., 1 8.5 Fr., 2 7 Fr. were placed to ameliorate symptoms of pain and recurrent episodes of pancreatitis. Four patients did not experience relief of symptoms, while 1 did. This failure to respond to decompression prompted further manipulation of the pancreatic duct, and the retrieved stents were examined for tumor.

### METHODS

After removing the stent from the duct in which it was placed, saline is flushed through the stent. The effluent is spun down by centrifugation, and the sediment examined by a histocytologist. The stents are examined for evidence of gross tissue, and an attempt is made to remove or extricate debris from the side holes or flaps. If tissue is found, it is fixed, stained, and examined microscopically for malignant cells. If no malignant cells are evident, the stent is split longitudinally and the inner surface is scraped of all debris (Fig. 1). The debris is then placed on filter paper (Fig. 2) and processed in the usual and standard manner for fixation and stainings, and the slide is examined (Fig. 3 and 4).

### RESULTS

Twelve of 16 samples obtained from stents were positive for adenocarcinoma; 3 of 5 from the pancreas and 9 of 11 from the bile duct. Cytologic specimens were positive in only 4 (3 bile duct, 1 pancreas) of the 16 specimens analyzed. The diagnosis of adenocarcinoma was confirmed at surgery in the remaining 4 patients (Table 1).

### DISCUSSION

Although cholangiographic appearances of strictures of the bile duct and pancreas are predictive of a malignant etiology in 90% of cases (Nix *et al.*, 1988; Snady *et al.*, 1992; Siegel, 1991), definitive treatment, i.e. chemotherapy or radiation, usually is initiated only after tissue confirmation of the malignancy has been made, with few exceptions. Some benign conditions such as sclerosing cholangitis and choledochal cysts are associated with an increased risk of malignant transformation that is extremely difficult to detect in its earlier stages unless a high index of suspicion prompts an aggressive approach to tissue sampling.

Various modalities have been used to obtain tissue. Exfoliative cytology of bile aspirate or pancreatic juice collected after secretin stimulation has a sensitivity of 34–79% as reported by several groups (Muro *et al.*, 1983; Kawai *et al.*, 1987; Harada, 1979. Endo *et al.*, 1974; Hatfield *et al.*, 1976; Roberts-Tomson and Hobbs, 1979). Tissue sampling for examination can be obtained by: 1) direct endoscopic biopsy obtained from a visible lesion e.g., ampullary carcinoma; 2) from within the bile duct at the time of ERCP cholangiography by using fluoroscopic guidance, or 3) during direct cholangioscopy either with the daughter scope at ERCP or a cholangioscope that is advanced through a percutaneous tract (Siegel, 1991; Rustgi *et al.*, 1989). Percutaneous fine needle aspiration of masses performed under CT or sono guidance requires identification of a mass lesion. The success rate for obtaining tissue and confirming malignancy using needle aspiration techniques approaches 70–80% in cases of carcinoma of the head of the pancreas. Results are much lower for cholangiocarcinoma and small pancreatic tumors (Ferrucci *et al.*, 1980; Teplick *et al.*, 1988). A second method, the Trucut[R] biopsy gun, is also utilized under CT/sono guidance providing core tissue that is adequate for diagnosis (Parker *et al.*, 1989). Both these percutaneous techniques carry a low but definite risk of seeding the needle tract with tumor cells (Ferrucci *et al.*, 1979; Smith *et al.*, 1980).

Brushing biopsy methods were first described by Weidenhiller and Osnes (Anaker *et al.*, 1977; Osnes *et al.*, 1975). Brushing samples obtained when using a conventional cytology brush have been reported to yield positive results ranging from 18–52% Rustgi *et al.*, 1989; Osnes *et al.*, 1975; Ryan, 1989; Venu *et al.*, 1989. The low yield of this method is due to 1) inaccessibility of the lesion; and 2) desmoplastic changes that occur with certain lesions such as cholangiocarcinoma. Certainly, one can not expect to obtain positive brushings from a bile duct that is compressed by an extrinsic tumor mass, as seen in metastatic carcinoma or carcinoma of the head of the pancreas, until there is luminal involvement or invasion of the duct. Technically, it may be difficult to advance a conventional brush into the stricture. A guidewire system

**Table 1**

| Patient Age | Gender | Stricture | Location | Scrape Results | Cytology |
|---|---|---|---|---|---|
| 57 | F | Bile duct | Klatskin III | Negative | Positive |
| 63 | M | Bile Duct | Klatskin III | Positive | Negative |
| 73 | M | Bile Duct | Klatskin III | Negative | Negative |
| 77 | M | Bile Duct | Klatskin II | Positive | Negative |
| 83 | F | Bile Duct | Klatskin II | Positive | Negative |
| 48 | F | Bile Duct | Klatskin II | Positive | Positive |
| 59 | M | Bile Duct | Klatskin II | Positive | Negative |
| 84 | F | Bile Duct | Klatskin I | Positive | Negative |
| 69 | F | Bile Duct | Klatskin I | Positive | Negative |
| 75 | M | Bile Duct | Klatskin I | Positive | Negative |
| 51 | M | Bile Duct | Klatskin I | Positive | Positive |
| 76 | M | Pancreas | Head | Negative | Negative |
| 47 | F | Pancreas | Head | Positive | Negative |
| 49 | F | Pancreas | Head | Negative | Positive |
| 63 | M | Pancreas | Genu | Positive | Negative |
| 67 | M | Pancreas | Genu | Positive | Negative |

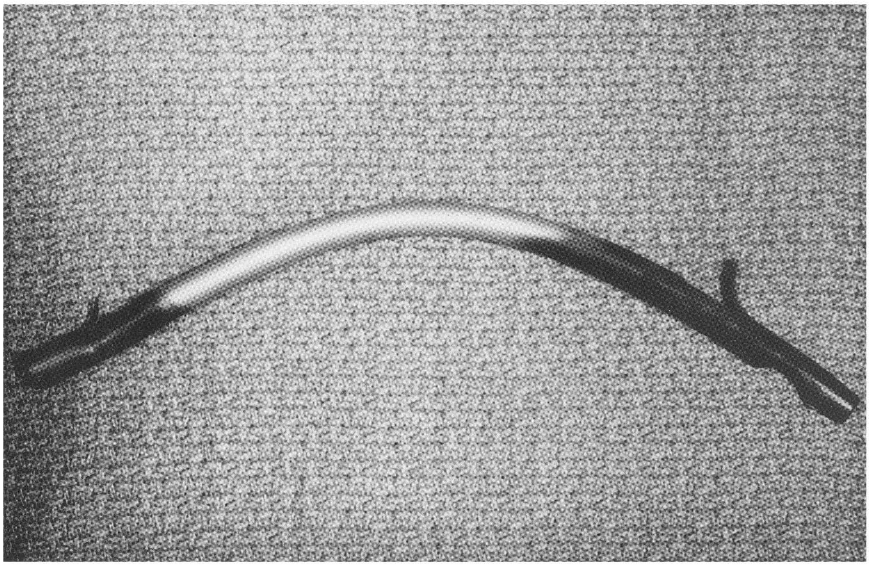

**Figure 1** Large caliber prosthesis removed from bile duct. Note staining of duodenal portion exposed to intestinal juices.

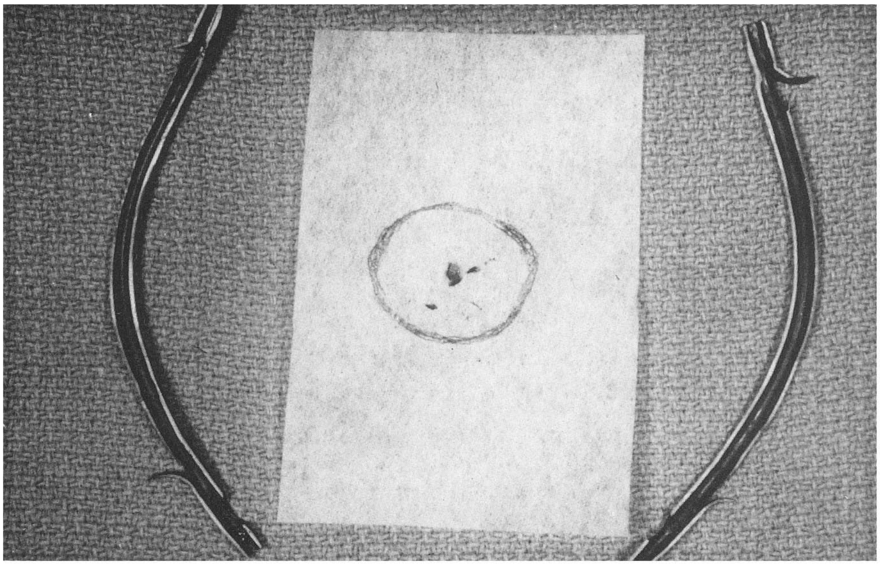

**Figure 2** A composite picture of transected stent and material scraped from lumen placed on filter paper.

would facilitate sampling. Foutch, *et al.*, described a technique using a conventional cytology brush passed alongside a guidewire, and this technique yielded a diagnostic sensitivity of 56% (Foutch *et al.*, 1989). In two other reports, the authors used a new cytology brush that yielded a diagnostic sensitivity of 50% (Scudera *et al.*, 1990) and 70%, respectively. However, despite the low yield, the authors reported a specificity of 100% in both series (Scudera *et al.*, 1990; Venu *et al.*, 1990).

Brushings of biliary strictures can also be obtained percutaneously using either a cytology brush and/or scraping device passed over a guide wire. This combined technique has been reported to yield a positive result of 60% Yip *et al.*, 1988; Mendez *et al.*, 1980.

Peroral choledochoscopy using the mother-daughter endoscope system can facilitate biopsy and cytology specimens obtained by direct vision, but technical difficulties commonly associated with this technique prohibit a yield

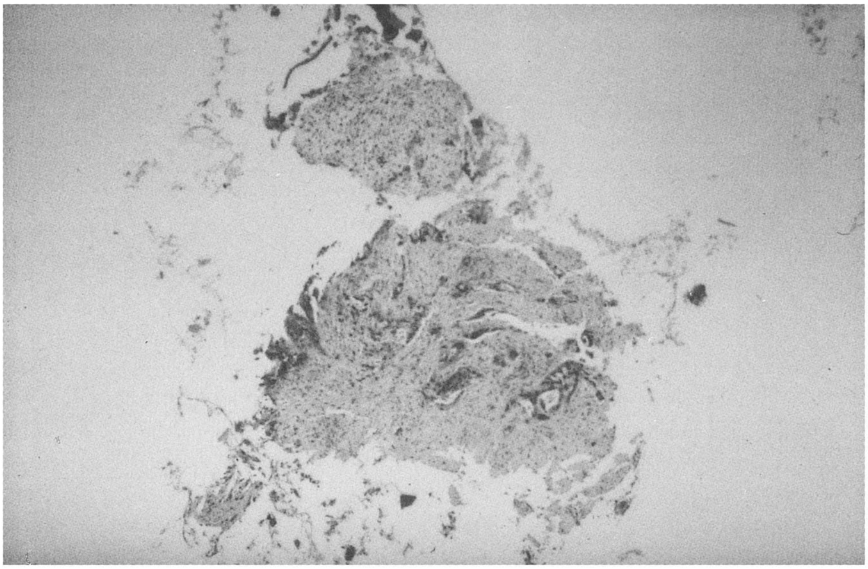

**Figure 3** Low power view of solid tissue with H & E stain.

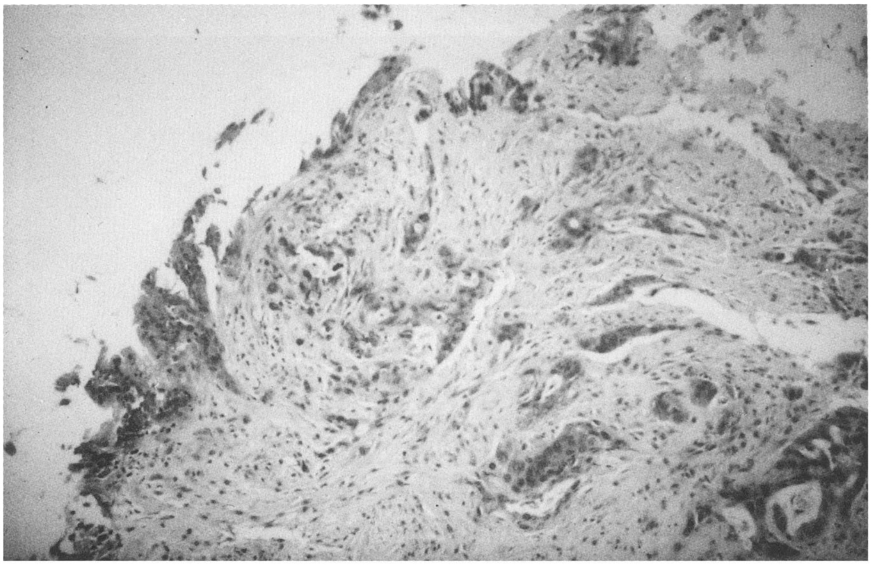

**Figure 4** High power view of adenocarcinoma.

higher than other methods. Many centers are using the mother-daughter system, and, after acquiring further experience, a higher yield is expected with this method (Siegel, 1991).

Leung reported in a letter that, after obtaining samples from the material embedded near the upper flap of stents removed from patients with presumed malignant obstruction, histologic examination did indeed reveal carcinoma in 11 of 14 patients he studied (Leung *et al.*, 1989).

The method we describe here is simple and practical. Our continuing experience using this method to evaluate stents retrieved from the bile duct has shown that not only has the diagnostic yield remained high, but no false-positive results have been obtained. The same method has been applied to pancreatic stents retrieved from 5 patients in whom the differential diagnosis included idiopathic pancreatitis or carcinoma. Although there is some time delay in establishing a diagnosis (waiting 2 to 4 months for the stent to occlude), 2 cases studied in this series yielded positive results as early as 72 hours after placement of the prostheses. In highly suspicious lesions, it may be advisable to place a prosthesis, after having obtained cytology and biopsy specimens, and advising the patient to return in a week for removal of the prosthesis for analysis. Most strictures require therapy of obstructive lesions, and are not necessarily a prerequisite for this technique. This technique, then, is complimentary to decompression techniques.

In conclusion, we believe that the method we discuss above for obtaining tissue samples from the bible duct and pancreas is 1) simple, 2) useful, 3) specific, and 4) should become an adjunct to biopsy and brush cytology. When stent scraping is combined with endoscopic biopsy and cytology techniques, higher positive yields are expected. We routinely send stents and washings from stents obtained from all patients in whom the index of suspicion is high and encourage others not to discard the retrieved stents since the histologic prognosis may be present within the stent.

### Case 1

Y. S. was a 76-year-old white male with a history of chronic pancreatitis. He presented with painless jaundice and an enlarged liver. A CT scan showed a focal increase in the size of the head of the pancreas, but a CT guided biopsy of the pancreas was negative for malignancy. An ERCP was performed and revealed a stricture in the head of the pancreas. Brushings were obtained from the stricture, and a large caliber stent was placed into the pancreatic duct. The brushings obtained at ERCP were negative for malignant cells. His obstructive symptoms were relieved by the stent, but 4 months later, they recurred. An ERCP was repeated on readmission, and the stent was found to be occluded. The stent was removed, placed in formalin for fixation, and later examined for malignant cells. The cytology showed a moderately well-differentiated adenocarcinoma of the pancreas.

### Case 2

M. F., a 51-year-old white male, had undergone a cholecystectomy and choledochoduodenostomy for stone disease. Six months later, he complained of nausea, weight loss, and upper abdominal pain. On physical examination, his liver was mildly enlarged and tender His liver chemistries and serum amylase levels were elevated. A CT scan and ultrasound revealed a dilated common hepatic duct, but no lesions were seen in the liver or pancreas. An ERCP was performed, revealing a stricture of the distal common bile duct with proximal bile duct dilatation. Brushings obtained from the stricture at ERCP were negative for malignancy. A stent was placed which relieved his symptoms, but, 2 months later, his obstructive symptoms recurred and an ERCP was repeated. The stent was found to be occluded, and it was removed and placed in formalin. Scrapings from the stent were positive for adenocarcinoma.

### REFERENCES

Anaker H., Weiss H. D., Kramann B., eds. (1977) Endoscopic retrograde pancreaticocholangiography. Berlin, Springer-Verlag.

Endo Y., Takeshi Tamura H. *et. al.* (1974) Cytodiagnosis of pancreatic malignant tumors by aspiration under direct vision using a duodenal fiberscope. *Gastroenterology*, 67:944–951.

Ferrucci J. T., Wittenberg J., Margolis M. N. *et. al.* (1979) Malignant seeding of pancreatic cancer by skinny-needle aspiration biopsy. *Radiology*, 130:345–346.

Ferrucci J. T., Wittenberg J, Mueller P. K. *et. al.* (1980) Diagnosis of abdominal malignancy by radiologic fine-needle aspiration biopsy. *Am J Roentgenol*, 134:323–330.

Foutch P. G., Harlan J. R., Kerr D. *et. al.* (1989) Wire-guided brush cytology: A new endoscopic method for diagnosis of bile duct cancer. *Gastrointest Endosc.* 35:243–247.

Harada H. (1979) Collection of pancreatic juice and bile via duodenoscope for cytodiagnosis and exocrine pancreatic function test. In: Takemoto T., Kasugi T. (eds.): *Endoscopic retrograde Cholangiopancreatography*. Igaku-Shoin, Tokyo. 58–77.

Hatfield A. R., Smithies A., Wilken R. *et. al.* (1976) Assessment of ERCP and pure pancreatic juice cytology in patients with pancreatic disease. *Gut*, 17:14–21.

Kawai K., Yasuda K., Nakajima M. (1987) Endoscopic diagnosis of cancer of the pancreas. In: Sivak MV, ed. Gastroenterologic *Endoscopy*. WB Saunders, Philadelphia. 821–838.

Leung W. C., Sung J. Y., Chung S. C. S., *et. al.* (1989) Endoscopic scraping biopsy of malignant biliary strictures (letter). *Gastrointest Endosc*, 35:65–66.

Mendez G., Russell E., Levi J. U. *et. al.* (1980) Percutaneous brush biopsy and internal drainage of the biliary tree through endoprosthesis. *Am J Roentgenol* 134:653–659.

Muro A., Mueller P. R., Ferrucci Jr. J. T. *et. al.* (1983) Bile cytology: a routine addition to percutaneous biliary drainage. *Radiology*, 149:846–847.

Nix G. A., Van Overbeeke I. C., Wilson J. H. *et. al.* (1988) ERCP diagnosis of tumors in the region of the head of the pancreas: analysis of criteria and computer aided diagnosis. *Dig Dis Sci*, 35:577–586.

Osnes M., Sirck-Hanssem A., Myren J. (1975) Endoscopic retrograde brush cytology (ERBC) of the biliary and pancreatic ducts. *Scand J Gastroenterol.* 10:829–831.

Parker S. M., Hopper D, Yakes W. F. *et. al.* (1989) Image-directed percutaneous biopsies with a biopsy gun. *Radiology*, 171:663–669.

Ryan E. (1989) Cytologic brushings of ductal abnormalities identified during ERCP. *Gastrointest Endosc.* 35:168 (abstract).

Roberts-Tomson I. C., Hobbs J. B. (1979) Cytodiagnosis of pancreatic and biliary cancer by endoscopic duct aspiration. *Med J Aust.* 1:370–372.

Rustgi A. K., Kelsey P. B., Guelrud M *et. al.* (1989) Malignant tumors of the bile ducts; diagnosis by biopsy during endoscopic cannulation. *Gastrointest Endosc.* 35:248–251.

Scudera P. L., Koizumi J., Jacobson I. M. (1990) Brush cytology evaluation of lesions encountered during ERCP *Gastrointest Endosc*, 36:281–284.

Siegel J. H. (1991) Endoscopic retrograde cholangiopancreatography: Technique, Diagnosis, Therapy. Raven Press, New York.

Smith F. P., MacDonald J. S., Schein P. S. *et al.* (1980) Cutaneous seeding of pancreatic cancer by skinny-needle aspiration biopsy. *Arch Intern Med.* 140:855.

Snady H., Siegel J. H., Cooperman A. M. (1992) Clinical effectiveness of endoscopic ultrasound compared with computed tomography and ERCP in obstructive jaundice or small peripancreatic mass. *Gastrointest Endosc*,. 38:27–34.

Teplick S. K., Haskin P. H., Kline T. S. *et. al.* (1988) Percutaneous pancreaticobiliary biopsies in 173 patients using ultrasound or fluoroscopic guidance. *Cardiovasc Intervent Radiol*, 11:26–28.

Venu R. P., Geenen J. E., Kini M. *et. al.* (1990) Endoscopic retrograde brush cytology: A new technique. *Gastroenterol*, 99:1475–1479.

Venu R. P, Prabhu M., Geenen J. E. *et. al.* (1989) Improved sensitivity in the cytologic diagnosis of cholangiocarcinoma. *Gastrointest Endosc.* 35:181 (abstract).

Yip K. Y., Leung J. W. C., Chan K. M., *et. al.* (1988) Scrape biopsy of malignant biliary strictures through percutaneous transhepatic biliary drainage tracts. *Am J Roentgenol*, 152:529–530.

